# Electrosensitivity in planthoppers (Insecta: Hemiptera: Auchenorrhyncha: Fulgoromorpha)

**DOI:** 10.1007/s00359-025-01790-1

**Published:** 2026-01-07

**Authors:** Peter Bräunig, Hannelore Hoch, Werner Baumgartner

**Affiliations:** 1 Institute Biology II, RWTH Aachen University, Worringerweg 3, 52056 Aachen, Germany; 2 Leibniz-Institute for Evolution and Biodiversity Science, Center for Integrative Biodiversity Discovery, Museum für Naturkunde Berlin, Invalidenstr. 43, 10115 Berlin, Germany; 3 Senckenberg Deutsches Entomologisches Institut, Eberswalder Str. 90, 15374 Müncheberg, Germany; 4 Institute of Biomedical Mechatronics, Johannes Kepler University Linz, Altenberger Straße 69, Linz, 4040 Austria

**Keywords:** Insect, Fulgoromorpha, Electric fields, Sensory physiology, Sensory pits

## Abstract

Extracellular recordings from the sensory pits, conspicuous sensory organs on the cuticle of planthopper nymphs (suborder Fulgoromorpha), were performed. No responses to sound, ultrasound, direct mechanical stimulation, temperature changes, or magnetic fields were observed. They do, however, respond to stimulation with electric fields of very low intensity. Field strengths of less than 1 kV/m were sometimes sufficient to elicit responses. These responses, together with the arrangement of these special sensory structures on the body surface of planthoppers, imply that these sensory organs perceive electric fields in the environment. Our results correspond well with recently published observations and model calculations for treehoppers (Membracidae), another Auchenorrhyncha taxon. Our results corroborate these recent findings with direct electrophysiological evidence and support the notion that both treehoppers and planthoppers are able to perceive electric fields. The ecological importance for this kind of sensory system is discussed.

**Supplementary Information:**

The online version contains supplementary material available at 10.1007/s00359-025-01790-1.

## Introduction

A recently published paper presents observations and modelling results which demonstrated that treehoppers (Membracidae) are probably able to perceive electric fields, and that this may be facilitated by the special, and in some species rather bizarre, morphology of the pronotum in this group of insects (England et al. 2025). These authors also looked for sensilla that might be able to convey this modality, and provided evidence that “pit-type setae” on the pronotum could be well suited for this task. They described these as “shorter setae protruding from near to the rim of pits in the cuticle and projecting across some or all of the diameter of the pit”. The diameter of these pits varies between 10 and 70 μm in the species they investigated (values estimated from Fig. 5 in England et al. 2025).

**Fig. 1:**
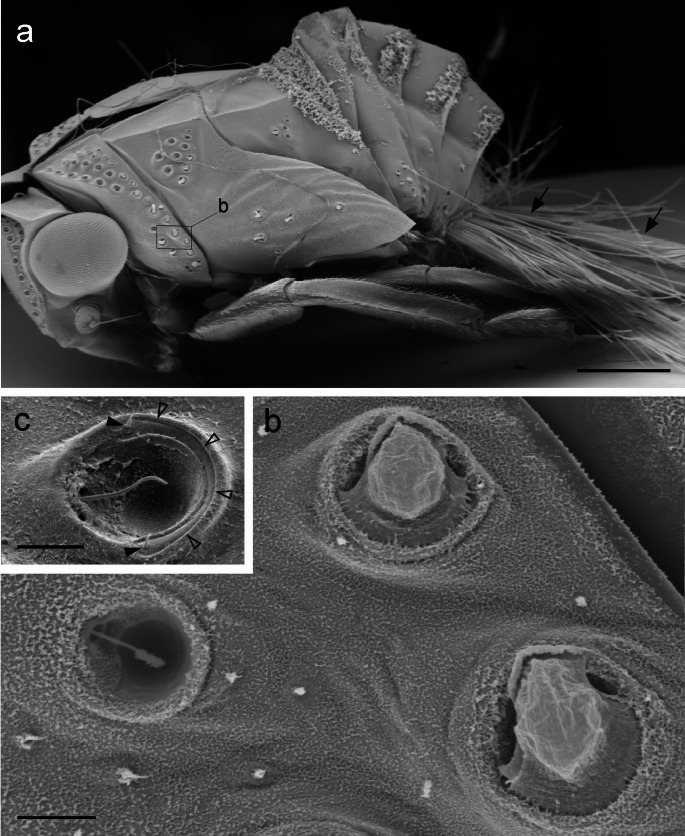
Sensory pits of *Issus coleoptratus*. **a** Exuvia of a fifth nymphal stage showing the pits on the head and thoracic tergites. Arrows indicate wax threads emerging from glands at the tip of the abdomen. **b** A small region of the pronotum (indicated by rectangle in **a**) showing three pits, two on the right with their cupolae intact, one on the left without cupola. **c** A single pit of a nymph after treatment with organic solvents. The base of the cupola is visible as a crescent (open arrowheads). Two short setae (black arrowheads) are visible near the cusps of this crescent. Scales: 500 μm in **a**, 20 μm in **b**, 10 μm in **c**

**Fig. 2:**
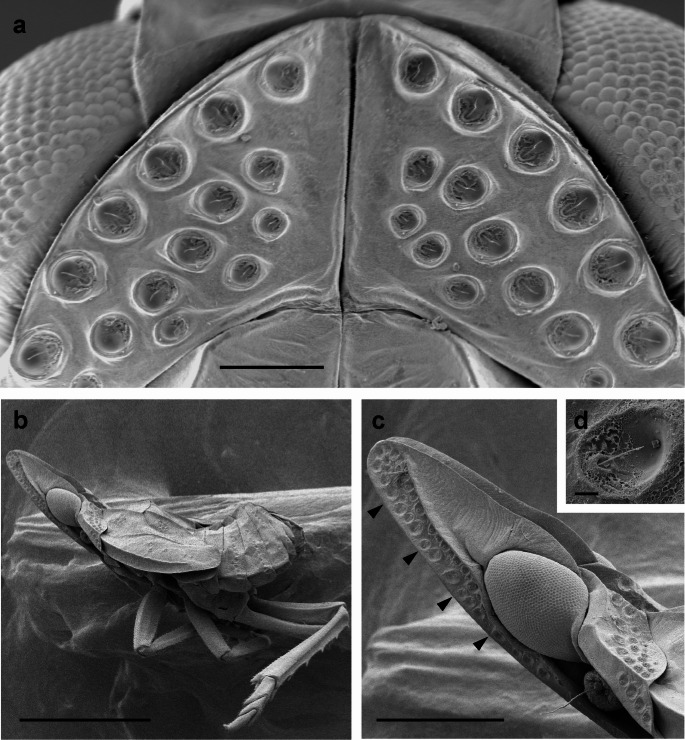
Sensory pits of final instar nymphs of *Issus coleoptratus* and *Dictyophara europaea* **a** Dorsal view of the pronotum of *I. coleoptratus* (anterior to the top). Please note the strict bilateral symmetry of the orientation of the setae within the pits. For each pit on one side one can find a corresponding pit on the contralateral side with mirror image morphology. **b** Fifth instar nymph of *D. europaea* with sensory pits on the head and thoracic and abdominal tergites. Please note the elongated head, that is very large in relation to the body. **c** Head and pronotum at higher magnification showing the dense array of sensory pits on the head (arrowheads). **d** A single sensory pit. Please note the absence of a cupola and additional short setae (compare Fig. 1c). Scales: 100 μm in **a**, 2 mm in **b**, 0.5 mm in **c**, 10 μm in **d**

The pit-type setae investigated by England et al. (2025) share many morphological similarities with the previously described sensory pits found in nymphs of all planthopper taxa at the family rank (Fulgoromorpha, except for the Tettigometridae and Hypochthonellidae; Asche 1985; Yang and Yeh 1994; Emeljanov 2001). In these groups the sensory pit consists of a bowl-shaped depression in the cuticle into which, from the rim of the bowl – and, in some species, out of a conspicuous socket (Figs. 1b, c and 2d)—a sensory seta protrudes. Unlike most mechanoreceptive sensory setae in insects, these structures do not stand out perpendicular to the body surface. Positioned more or less level with the body surface, they span the pit for about two thirds of its diameter, and are often slightly bent (Figs. 1 and 2).

These pits occur in all of the five nymphal stages and, in rare cases, also in adult individuals of certain families, namely Achilixiidae, Meenoplidae, Cixiidae (tribe Bennini Metcalf, 1938 and tribe Bennarellini Emeljanov, 1989) and Flatidae. In nymphs, sensory pits are most abundant on the head and pronotum, but they also occur on thoracic and abdominal tergites, and on the wing buds. They are, with only one known exception (Gnezdilov and Wilson 2007), restricted to the dorsal and lateral regions of the body (Figs. 1 and 2). Their morphology was described in detail in the planthopper *Issus coleoptratus* (Issidae; Bräunig et al. 2012). Here the pits vary from 20 to 70 μm in diameter. Size, position, and, remarkably, the orientation of the large setae within the pits follows a strict mirror image symmetry on the different parts of the body, and differs only slightly when different specimens at the same nymphal stage are compared (Fig. 2a; Bräunig et al. 2012). This almost invariant orientation of the setae within the pits, their “polarity” as it were, suggests that the entire system is best suited to perceive a field-like stimulus, and that earlier speculations about their function are probably incorrect. Sulc (1928), the first author to describe these structures, regarded them as hygroreceptors without providing any evidence for this notion. Liebenberg (1955) discussed their possible function as proprioceptors, hygroreceptors, or vibration sensors, but already listed numerous arguments against all three assumptions.

Following our detailed morphological analysis, we began to study the physiology of the sensory pits in nymphs of *Issus coleoptratus* in order to elucidate their function (adults of this species no longer have sensory pits). Much to our surprise, the only modality the sensory pit afferents responded to were electric fields. They also reacted very sensitively to this modality, however, our experiments failed to elicit a specific behavioural response from the nymphs. Our results, however, substantiate previous results reported by England et al. (2025) by providing direct evidence for electrosensitivity by electrophysiological experiments.

## Materials and methods

### Insects

Final instar *Issus coleoptratus* (Issidae) nymphs were collected from dense ivy growth (*Hedera helix*) in the garden of PB in Aachen (50.775 N, 6.061E) or on the university campus in Linz (48.338 N, 14.317E). Nymphs at this stage are available from late April to early May each year. They were kept on potted ivy plants or freshly cut ivy stems placed in glass vials filled with tap water. Such stems remain viable for extended periods and often develop roots. They are readily accepted as food plants by the nymphs. Last instar nymphs of *Dictyophara europaea* (Dictyopharidae) were collected during June 2019 in the vicinity of Dragonja, Slovenia (45.448 N, 13.664E). In the laboratory they were sustained on bind weed (*Convolvulus* sp.). For some control experiments a few specimens of *Schistocerca gregaria* (Orthoptera: Acrididae) from commercial stock origin were used (https://www.terraristikshop.net).

### Scanning electron microscopy

Images were made from either air-dried exuviae or nymphs preserved in 70% alcohol. The latter were dehydrated in an ascending alcohol series, rinsed in acetone and two changes (several hours each) in hexamethyldisilazan (HMDS). They were transferred to a fume cupboard to allow the HMDS to evaporate. Dried insects were mounted on stubs, sputtered with gold, and examined with a scanning electron microscope (REM 525 M; Philips AG).

### Electrophysiology

For electrophysiological experiments, nymphs were anaesthetised by cooling on ice and fixed on a small metal stand with modelling clay. After recording, the clay was carefully removed and in most cases the nymphs survived the whole procedure unharmed. Smaller nymphs in earlier stages did not survive the residual heat produced by the illumination, although we used cold light sources with heat filters. For recording we used electrolytically sharpened Tungsten wires (Goodfellow W 005138/14, 0.1 mm diameter). These wires were crimped into 23 gage cannulae together with a silver wire that could be soldered to a standard cable. The cannulae were fitted to suitable syringes mounted on a micromanipulator (type M33). A 10 μm silver wire inserted into the abdomen of the insect served as reference electrode. The Tungsten electrodes were gently pushed through the cuticle close to the conspicuous sockets of the large setae of the pits. In this fashion we achieved good recordings in about one fifth of all attempts. Signals were amplified with a WPI DAM 50 differential pre-amplifier and recorded using a CED 1401 micro interface and Spike 2 software (Cambridge Electronic Design, Cambridge, UK). Regions of interest from Spike 2 files were transferred to Canvas^®^ graphics software for further processing. Canvas^®^ and Libre Office Draw were used to prepare all figures.

The following stimuli were used during recording from sensory pits (summarized in Table 1): *Sound*: We used a loudspeaker connected to a function generator (Wavetek 182 A). We tested frequencies ranging from 50 Hz to 20 kHz. *Ultrasound*: Stimuli were generated by jangling a bundle of keys at different velocities in close vicinity to the insect. Jangling keys produces broad band noise way into the ultrasonic range (Gerhardy 2009).

**Table 1:** Test stimuli for sensory pit afferents

Modality	Device(s)	Parameters	Distance	Response
sound	speaker connected to frequency generator	frequency sweeps 50 Hz – 20 kHz, approx. 50–80 dB SPL	10–30 cm	no
ultrasound	key bundle	vigorous jangling	10–30 cm	no
magnetic fields	small pot magnets moved back and forth	pull strength 30–50 N	5–30 cm	no
gravity	tilt stage for insect plus manipulator with electrode	rotation through 90° from horizontal to vertical		no
direct movement of large setae in pit	Piezo crystal, manipulator	rotation of seta around its socket, 1–10°		no
temperature	small soldering iron	60–70 °C	as close as possible	no
temperature	1 mm diameter steel rod out of freezer	≥ −18 °C	as close as possible	no
plant volatiles	ground up ivy leaves		as close as possible	no
electric fields	PVC or glass rods	charged by friction	0.5–3 mm	yes
electric fields	silver wire connected to frequency generator	DC stimuli with sinusoidal, triangular, or rectangular modulation, 0.2–40 Hz, 1–20 V peak-to-peak	0.5–3 mm	yes

*Magnetic fields*: Small, flat pot magnets of 19 mm diameter (pull strength 30 N) or 29 mm diameter (pull strength 50 N) (bases of small magnetic stands; Hoffmann Supply Chain GmbH) were moved by hand and, in relation to the insect, either anterior to posterior, or up and down, thereby slowly reducing the distances from 30 to 5 cm. *Gravity*: We mounted the stand with the insect and the manipulator with the electrode on a metal plate fixed to a wooden board. This board was connected to a second one with hinges that allowed for a maximum tilt of 90°. By slowly and cautiously tilting by hand, in two experiments we succeeded to tilt the insects by 90° without losing the recording. *Direct mechanical stimuli*: A sharpened Tungsten wire was connected to a piezo crystal (RS PRO vibration sensor, Conrad Electronics). Into the crystal we fed sinusoidal or ramp stimuli generated by the function generator mentioned above and amplified by a DC amplifier (HP 6824 A) to produce larger movement amplitudes. The tip of the Tungsten wire touched the underside of the seta we recorded from. In this way the seta was lifted upwards by the movement. Values for movement angles were estimated. Because the seta moved towards the observer exact values could not be determined. Since the stimulations with the Piezo crystal did not elicit responses, larger movements were induced by turning the appropriate dial of the manipulator. Visible movements of the pit setae were also induced by air puffs applied via a Pasteur pipette. *Temperature*: Pits from which recordings were taken were approached with the tip of a small soldering iron heated up until hot to the touch. Alternatively, we approached the pits with a thin steel rod removed from a −18 °C freezer immediately before use. *Chemicals*: Pits were exposed to volatiles from freshly minced ivy leaves. Ivy leaves were folded, rolled between two fingers, and held as close as possible to the insect. We smelled intense plant odour immediately so we were confident, that the pits tested were also exposed. *Electric field stimulation*: During initial experiments, pit afferents were stimulated by manually moving back and forth glass or plastic rods, electrostatically charged by friction, at distances to the insect between 0.5 and 3 mm. By rubbing the rods against wool, glass rods carried positive charges and plastic (PVC) rods negative charges. For reliable control and the possibility to quantify the response to electric fields, in following experiments a small, time-variant voltage between a reference potential and a wire in the vicinity of the sensory pits was applied. Special care was taken to not touch the animal with the stimulation electrode, thus only the electric field through the air-gap could cause a response. We used 50 μm silver wires connected to a function generator (Wavetek 182 A). The wires were positioned parallel or perpendicular to the longitudinal body axis of the insect at a distance that varied between 0.5 and 3 mm. Sinusoidal stimuli (0.2–40 Hz) were applied with voltages between 1 and 20 V peak-to-peak.

**Fig. 3:**
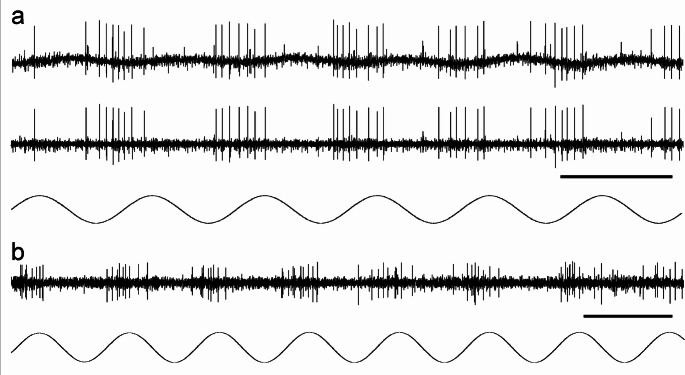
Recording from sensory pit neurons of final instar nymphs of *Issus coleoptratus* (**a**) and *Dictyophara europaea* (**b**). In both recordings a wire was positioned in the air and near the head approximately 1 mm distant from the pit and connected to a signal generator producing a sinusoidal voltage of 2 V (peak to peak) at 2 Hz. The pits recorded from were located on the tergum of the mesothoracic segment in both insects. The upper trace in **a** shows the original recording, the second trace the same after the application of a digital high pass filter (cut-off frequency 100 Hz; see text for details). The same filter was applied to the upper trace in **b**. Time scales: 0.5 s

**Fig. 4:**
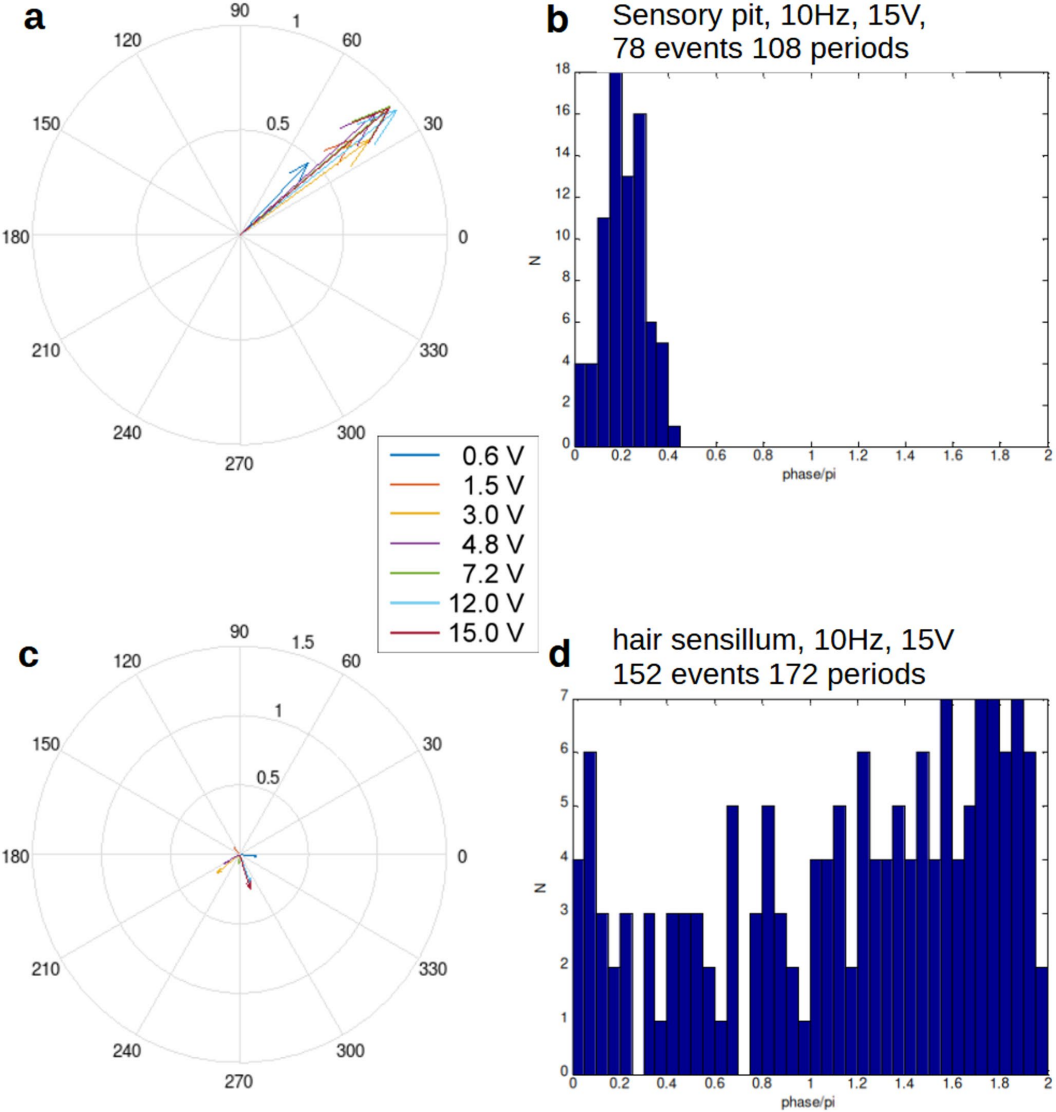
Phase-dependence at 10 Hz of spiking-response of sensory pit neurons (**a**, **b**) and neurons belonging to hair sensilla on the cuticle (**c**, **d**) of *Issus coleoptratus*. The phase-alignment of the spikes is shown in compass-diagrams (**a**, **c**) for different stimulation strengths and, as examples, for 15 V-stimuli as histograms (**b**, **c**)

In many recordings we observed direct crosstalk from the stimulation wire to the recording electrode (Fig. 3a, Supplementary Fig. 1a). Crosstalk amplitude increased with stimulation intensity or when the stimulation wire was moved closer to the insect. For this reason, we high pass-filtered (cut-off frequency 100 Hz) the recording traces in order to aid threshold detection (see below). Such crosstalk was also observed during the following control experiments. In these, using the same technique and applying identical electric field stimulation, we recorded from hair sensilla as well as from campaniform sensilla on the head and thorax of *Issus coleoptratus*, and from mechanoreceptive setae on abdominal sternites of the locust *Schistocerca gregaria*. All these mechanoreceptors readily produced action potentials in response to touch or vibration but did not respond to electric field stimulation even at high intensities (Fig. 4c, d).

For the analysis of the electrophysiological recordings, action potentials were identified using a threshold detector. Their temporal position was compared with the periodic stimulation signal, allowing the phase position of the spikes to be determined. The distribution of the phase positions was visualised using histograms or compass diagrams. For the latter, each of the N action potentials in a recording was assigned a vector with length 1/N and angle φ (corresponding to the phase position). These vectors were summed and displayed as a vector arrow starting at the origin of a coordinate system. If perfect phase synchronisation were present, a vector arrow of length 1 would be expected at the corresponding angle; in the case of unsynchronised behaviour, the expected value would be 1/√n (as the mean square displacement has expected value 1/n) and the angle would be evenly distributed from 0 to π, or from 0 to 360° respectively. The import of the electrophysiological recordings as well as data analysis and plotting were performed using self-written Octave programmes.

### Conductance of the cuticle

Placing the tips of two metal electrodes onto the cuticle of *Issus* allowed us to measure the conductance between them. Due to the complicated geometry the specific conductance could not yet be determined.

### Behavioural experiments

*Issus* nymphs often remained in the same location on the ivy plants for hours, sometimes even days, so that for our behavioural investigations we assumed that they had adapted to the situation before we presented them with stimuli. For quick tests plastic or glass rods, electrostatically charged by friction, were manually moved back and forth close to the insects. In order to increase reproducibility and to avoid optic stimulation we stimulated the nymphs with charged wires using the stimulus regimes identical to those that had reliably elicited responses in the neurons innervating the setae of the sensory pits in the electrophysiological experiments. For these investigations 24 nymphs were tested.

## Results

### Electrophysiology

**Fig. 5:**
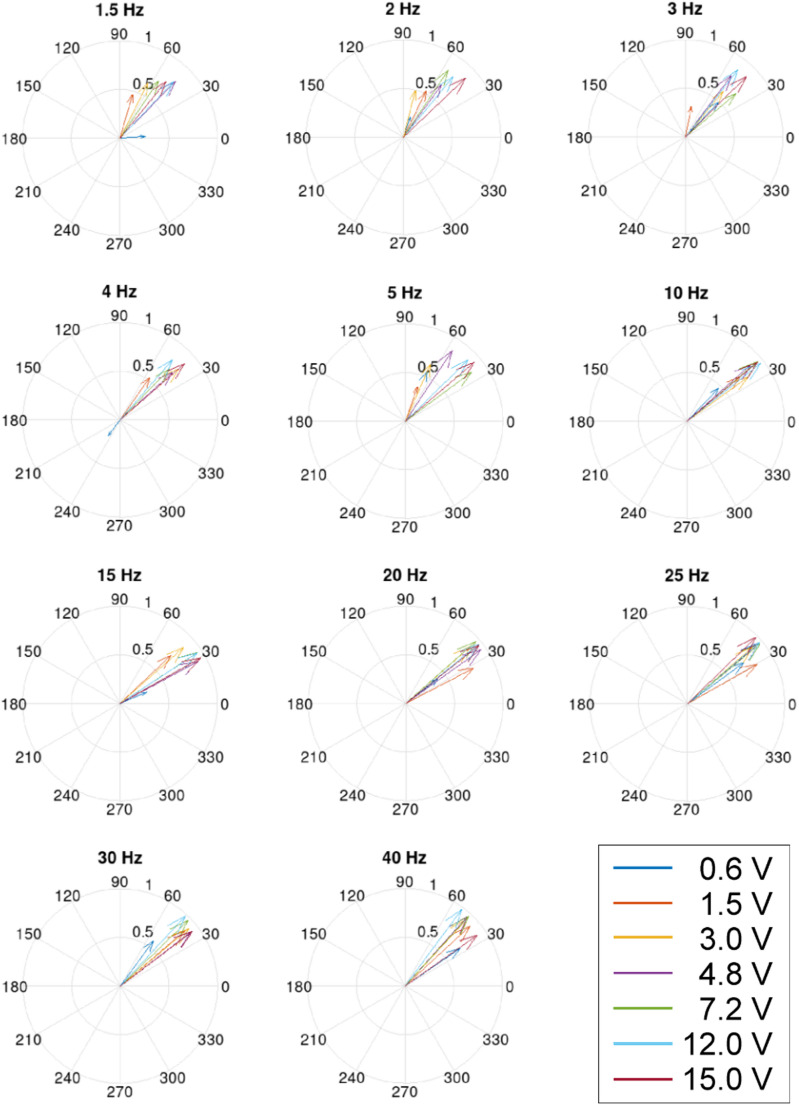
Phase-dependence of one sensory pit afferent at different stimulation frequencies ranging from 1.5 Hz to 40 Hz and for different stimulation voltages from 0.6 to 15 V

Sensory pit afferents did not respond to sound, ultrasound, light, temperature, green leaf scent, acceleration, or magnetic stimulation. Direct mechanical stimulation in rare cases elicited a response, but only when using large movement amplitudes. About 1 in 10 of the pit afferents reacted, but the pit setae had to be rotated by at least ten degrees to elicit responses. Likewise, air puffs did not cause any spikes. Changing the direction of the gravity vector during recording also had no effect. Sensory pit afferents only showed reactions when stimulated by electric fields (Figs. 3, 4 and 5, Supplementary Fig. 1, Table 1). We noticed this because electrostatically loaded plastic or glass rods moved back and forth close to the insect caused responses. Likewise, a charged wire in the vicinity of the sensory pit caused responses. The wire did not touch the animal, thus only the electric field through the air-gap could have caused them. The organs responded very sensitively and reliably to this kind of stimulus. For detailed analysis a sinusoidal voltage was applied to the stimulation electrode. Typically, phase-locked action potentials occurred when our stimulation wire was placed 0.5 to 3 mm away from the insect. Usually, stimulus intensities of only 1 V were sufficient to elicit responses (Figs. 3, 4 and 5). With these parameters the field strength would vary between 300 and 2000 V/m.

To quantify the response, especially with respect to the phase-lock, stimulation frequency and stimulation strength were varied systematically. In total, five sensory pits of *Issus coleoptratus* could be recorded from over the entire range of stimulation parameters and many others could be used for a partial frequency series. One example for the 10 Hz-stimulation is shown in Fig. 4, and the whole frequency-series of this particular sensory pit is depicted in Fig. 5. Clearly, over the whole frequency range, a phase lock, dependent on the signal strength, can be observed. This was also the case for all other sensory pits investigated. To rule out unspecific crosstalk-effects or simple mechanical stimulation by Coulomb-forces we also performed this stimulation protocol for mechanoreceptors (Fig. 4c, d). As is clearly evident, the sensory pits responded significantly phase-locked to the sinusoidal stimulation, whereas mechanoreceptors exhibit no significant phase lock. Also, the firing rate of the mechanoreceptors did not change with the electric field, neither for hair sensilla, nor for campaniform sensilla. Thus, the observed activity consists of spontaneous action potentials that are not correlated with the electric field stimulation; a clear contrast to the sensory pits.

The phase-lock effect was observed for a wide range of stimulation frequencies at different stimulation voltages as shown in Fig. 5. Here one single sensory pit was stimulated at various frequencies systematically. The compass diagrams allow for rapid judgement of the phase-lock effect. Clearly the effect increases with increasing stimulation strength. The stimulation strength appears to be the slope of the voltage over time, i.e. the faster the electric field changes the stronger the effect. The number of spikes per stimulation period increases at 1.5 Hz from 0.6 at 600mV to 2.5 at 15 V stimulation voltage. Thus, the pits clearly respond to changes in the electric field, i.e. the slope of the field strength over time.

This behaviour is not restricted to sinusoidal stimulation, but could also be observed when triangular or sawtooth-shaped stimulation voltages were applied (Supplementary Fig. 1).

When measuring the conductance between two electrodes, we found that the cuticle has isolating properties almost everywhere. When placing both electrode tips inside one of the pits, however, the resistance dropped to rather low values. Due to the complicated geometry the specific conductance could not yet be determined. It is evident, however, that the pits are ‘coated’ with a conductive material. This could shield the electric field when coming through the animal, yielding a certain directional sensitivity. Due to the setup available and measurement method, we were so far unable to characterise directionality of the sensory pits.

### Behavioural observations

Nymphs adapted to laboratory conditions were exposed to electric stimuli identical to the ones used for recording from the sensory pit afferents. Sinusoidal stimuli of frequencies ranging from 0.5 to 20 Hz and amplitudes up to 20 V and presented at different distances (minimal distance approximately 1 mm) did not elicit any visible, clear and reproducible response from any of the 24 nymphs tested. Likewise, approaching the nymphs with electrostatically loaded plastic or glass rods did not cause any clear and reproducible reactions.

## Discussion

In order to elucidate the function of the sensory pit organs of Fulgoromorpha nymphs, we tested several stimulus modalities: sound, ultrasound, magnetic fields, gravity, direct mechanical stimulation, temperature and electric fields (summarized in Table 1). Our electrophysiological experiments provide strong evidence that sensory pits perceive electric fields and, more specifically, are sensitive to temporal changes of external electric fields. Here we discuss our findings in the light of recent pioneering work on the electric sense of insects (Newland et al. 2008; Jackson et al. 2011; Clarke et al. 2013, 2017; Sutton et al. 2016; Khan et al. 2021; England and Robert 2024; England et al. 2025) and reflect on the relevance of electric sense for the insects´ ecology.

### Electrophysiology

In *Issus* nymphs, the sensory pits are covered by a bubble-shaped, fragile, translucent dome or cupola of presumably waxy material (Fig. 1b) that extends from the rim of the pit on the side opposite to the socket of the large seta (Bräunig et al. 2012). Into the base of this cupola protrude one or two additional minute setae. These additional setae as well as the crescent-shaped base of the cupola can be seen after treatment with organic solvents (Fig. 1c). The axons of all afferents from a single pit converge before entering the peripheral nerves (Bräunig et al. 2012) so that in principle in all recordings in this species we were never sure which afferent we recorded from. For this reason, we recorded from pits of *Dictyophara europaea*. In this species, as in perhaps most other species of the Dictyopharidae and Fulgoridae, the sensory pits have only the large setae stretching across the pit. Their pits lack cupolae and additional setae (Fig. 2c). The reactions of pit afferents to electrical stimuli were identical in this species (Fig. 3b). This makes us confident that we always recorded responses from the afferents of the large setae in both species and that it is the sensory neurons of these large setae that perceive electric fields.

The afferents did respond to direct movement of the setae, but only reluctantly. If they did respond, the movement amplitude had to be very high. While many insect mechanoreceptive setae are notoriously sensitive and respond to displacements of a fraction of a degree (Thurm 1983), the setae of the pits had to be moved by several degrees in order to elicit a response. This probably correlates with the highly modified tubular bodies in the dendritic tips of the sensory neurons of these setae (Bräunig et al. 2012). The tubular bodies are not in direct contact to the outer dendritic membrane as is normally the case in mechanoreceptive insect neurons (Thurm 1983; Keil 2012). This morphological feature and the residual low mechanosensitivity supports the notion that the setae of the pits derive from mechanoreceptive setae, but have been modified for a different function.

Our recordings provide strong evidence that their novel function is the perception of electric fields, more specifically to temporal changes of the external electric field. They also show that their sensory neurons react very sensitively to this stimulus. Field strengths of less than 1 kV/m may elicit responses. This corresponds well with values published for electrosensitive setae of caterpillars (England and Robert 2024). The pit afferents not only reacted to the stimuli provided by a charged wire. We observed that they also reacted strongly to approaching pieces of plastic or glass electrostatically loaded by friction. Thus, using their pits Issidae and Dictyopharidae can detect either (i) charged objects that are either approaching or moving away, depending on the charge state, or (ii) they can detect temporal changes of local charges or dipoles. Here (i) would correspond to the ability to detect flying animals which charge up electrostatically during flight like birds (Badger et al. 2015) or insects (England et al. 2025). By contrast, (ii) would render them capable of detecting temporal changes of plant electric fields or changes in the atmospheric potential gradient while moving through the host plant (Clarke et al. 2017).

In an earlier publication (Bräunig et al. 2012) we already hypothesised that the entire system of pits might be suited to respond to a field-like stimulus modality because not only the number and location of the pits in a particular body region, but also the orientation of the large setae within the pits is highly ordered and shows a strict bilateral symmetry (Fig. 2a). Moreover, even between different individuals of the same nymphal stage the size, number, and orientation of the pits in a particular body region varies only slightly (Bräunig et al. 2012). Up to now we were not able to investigate a potential directional sensitivity of single sensory pits. This would require electrophysiological examinations with a measuring system that is galvanically isolated from an external stimulation system, including common ground. The external stimulation system must also be able to create very defined spatial and temporal field conditions. Perhaps directionality is achieved using the entire system of pits. When a stimulus approaches from a certain direction many pits might respond, but with different strengths depending on distance, their orientation, and their location on the body surface. From these differences, the central nervous system might be able to calculate directional information. Further experiments are needed to clarify this point.

England et al. (2025) show that extreme body extensions in the Membracidae might aid the perception of electric fields. On these extensions they found what they called “erect-type” setae. Their “pit-type setae” were found in more proximal regions. By contrast, planthopper nymphs and adults show tendencies to place sensory pits into distal locations. First, the slim and elongated heads seen in planthoppers collectively referred to as lantern flies (e.g. Dictyopharidae) show similarities with anteriorly directed extensions of the pronotum in some membracid species. These elongated heads bear many sensory pits (Fig. 2). Sensory pits may also be located on lateral extensions of abdominal segments (Supplementary Fig. 2). This morphological feature was described in species of the families Achilixiidae (Wilson 1989) and Cixiidae, here in the tribes Bennarellini (Holzinger and Kunz 2006; Holzinger et al. 2013; Viegas and Ale-Rocha 2019, 2022) and Bennini (Hoch 2013). In the Bennini, the tendency to displace the sense organs laterally is carried to an extreme: In adults of this taxon, elongate, rod-like appendages protrude from the proximal portion of the abdomen (Supplementary Fig. 2b). At their distal tips these appendages bear sensory structures that share several morphological features with the sensory pits (Hoch et al. 2014). As these appendages are mobile, these sense organs might be actively pointed at stimuli of relevance to the insect such as approaching predators or parasitoids. Finally, it should be noted that the lateral body extensions of the Achilixiidae, Bennarellini and Bennini are not homologous, but have evolved independently (Hoch 2013; Holzinger and Kunz 2006). Apparently, selection has favoured the development of sensory pit-bearing body extensions, indicating the importance this sensory channel has for these insects.

### Relevance of the electric sense in treehoppers and planthoppers

So far *Issus* nymphs showed no obvious behavioural reactions to electric stimulation. This may simply be due to the fact that we never managed to apply the specific combination of stimulus frequency and intensity which in nature would elicit responses, or that the insects react to such stimuli only when the context is right. In treehoppers, England et al. (2025) demonstrated avoidance reactions to electric fields in about 50% of their trials. These authors also provided evidence that electric fields produced by flying predatory wasps might well be perceived by these insects.

The system of sensory pits in Fulgoromorpha might also play a role in the avoidance of predators and parasites such as birds, spiders, katydids, or parasitic wasps. In eyeless soil- or cave-dwelling species (Hoch et al. 2021, 2025), this function might be even more important because optic signals cannot be perceived. There are two known parasitic groups specialised on these insects (Waloff and Jervis 1987). A family of flies, the Pipunculidae, is specialised in infecting Auchenorrhyncha. They are small, about the size of a fruit fly, nevertheless they most likely charge up electrostatically during flight (England and Robert 2022). The second parasitic group is represented by small wasps of the family Dryinidae. Here only the males have wings and do not attack other insects. Only the females do, and intriguingly these females are wingless. Perhaps by approaching planthopper nymphs via walking they avoid the electrostatic charging caused by flight and in this way avoid detection. Since these parasitic insects are small, they can perhaps only overwhelm small nymphs, but most likely not the adult hoppers. This might explain why sensory pits are only found in nymphs of most planthopper species. On the other hand, the presence of sensory pits on prominent body extensions in adults of certain families (see above) indicates the need to detect electrically-charged sources also at this stage in certain taxa.

An electric sense could also play a role in interspecific interactions such as phoresy and insect-plant communication. Such a function has recently been demonstrated for plant mites (García-Robledo et al. 2025). Most Fulgoromorpha are very small insects, and many forms are brachypterous. For such insects, effective dispersal over long distances might be greatly enhanced by phoresy. Nymphs might detect approaching birds that have charged up during flight (Badger et al. 2015) and cling to their feathers for transportation. Observations of insect phoresy on birds are rare (Borges 2022; Tomás et al. 2018), but this might be due to the methodological difficulties involved. That electric cues may play a role in phoresy has recently been shown for nematodes (Chiba et al. 2023). Electroreception has been shown to play an important role in insect-plant interactions. Several taxa such as bees, bumblebees and hoverflies have been shown to detect the electrical fields around flowers, and use the information to identify rewarding flowers and to assess the availability of nutrients (for review see England and Robert 2022). It is therefore conceivable that planthopper nymphs receive clues from the electric fields of their host plants which hold information on the plants’ physiological state.

Further experiments are needed to elucidate the exact function(s) of this fascinating sensory system. It is a very conspicuous system of sensory structures and obviously an important one: Sensory pits are already present in planthopper fossils preserved in amber since the Lower Cretaceous (Shcherbakov 2007; Szwedo 2007; Emeljanov 2011). This sensory system thus apparently served its purpose for 145 million years. The sensory pits of the Fulgoromorpha constitute a fascinating yet vastly unexplored system. It can be expected that intensified research on this system will contribute significantly to our understanding of the sensory world of insects.

## Supplementary Information

Below is the link to the electronic supplementary material.

Supplementary Material 1

Supplementary Material 2

## Data Availability

No datasets were generated or analysed during the current study.

## References

[CR1] Asche M (1985) Zur Phylogenie der Delphacidae Leach, 1815 (Homoptera Cicadina Fulgoromorpha). Marburger Entomol Publikationen 2:1–910 www.zobodat.at/Asche1985

[CR2] Badger M, Ortega-Jimenez VM, von Rabenau L, Smiley A, Dudley R (2015) Electrostatic charge on flying hummingbirds and its potential role in pollination. PLoS One 10:e0138003. 10.1371/journal.pone.0138003 26421845 10.1371/journal.pone.0138003 PMC4589311

[CR3] Borges RM (2022) Phoresy involving insects as riders or rides: life history, embarkation, and disembarkation. Ann Entomol Soc Am 115:219–231. 10.1093/aesa/saab051

[CR4] Bräunig P, Krumpholz K, Baumgartner W (2012) Sensory pits - enigmatic sense organs of the nymphs of the planthopper *Issus coleoptratus* (Auchenorrhyncha, Fulgoromorpha). Arthropod Struct Dev 41:443–458. 10.1016/j.asd.2012.06.001 22750128 10.1016/j.asd.2012.06.001

[CR5] Chiba T, Okumura E, Nishigami Y et al (2023) *Caenorhabditis elegans* transfers across a gap under an electric field as dispersal behavior. Curr Biol 33:2668-2677.e3. 10.1016/j.cub.2023.05.042 37348502 10.1016/j.cub.2023.05.042

[CR6] Clarke D, Whitney H, Sutton G, Robert D (2013) Detection and learning of floral electric fields by bumblebees. Science 340:66–69. 10.1126/science.1230883 23429701 10.1126/science.1230883

[CR7] Clarke D, Morley E, Robert D (2017) The bee, the flower, and the electric field: electric ecology and aerial electroreception. J Comp Physiol A 203:737–748. 10.1007/s00359-017-1176-6 10.1007/s00359-017-1176-6 PMC5599473 28647753

[CR8] Emeljanov AF (2001) Larval characters and their ontogenetic development in Fulgoroidea (Homoptera, Cicadina). Zoosyst Ross 9:101–121

[CR9] Emeljanov AF (2011) A new genus and a new species of lanternflies of the subfamily Cladyphinae (Homoptera, Fulgoridae). Entomol Rev 91:484–489. 10.1134/S0013873811040105

[CR10] England S, Robert D (2022) The ecology of electricity and electroreception. Biol Rev 97:383–413. 10.1111/brv.12804 34643022 10.1111/brv.12804

[CR11] England SJ, Robert D (2024) Prey can detect predators via electroreception in air. Proc Natl Acad Sci USA 121:e2322674121. 10.1073/pnas.2322674121 38768327 10.1073/pnas.2322674121 PMC11161757

[CR12] England SJ, Palmer RA, O’Reilly LJ, Chenchiah IV, Robert D (2025) Electroreception in treehoppers: how extreme morphologies can increase electrical sensitivity. Proc Natl Acad Sci 122:e2505253122. 10.1073/pnas.2505253122 40690666 10.1073/pnas.2505253122 PMC12318218

[CR13] García-Robledo C, Dierick D, Manser K (2025) Electric transportation and electroreception in hummingbird flower mites. Proc Natl Acad Sci 122:e2419214122. 10.1073/pnas.2419214122 39869792 10.1073/pnas.2419214122 PMC11804690

[CR14] Gerhardy C (2009) Ultraschallerzeugende Mikrostrukturen für batte-rielose Fernbedienungen. Doctoral Thesis, RWTH Aachen

[CR15] Gnezdilov VM, Wilson MR (2007) A new genus and a new species of the tribe Mithymnini (Hemiptera: Fulgoromorpha: Nogodinidae) from Namibia, with sternal sensory pits in the adult. Zootaxa 1453:55–62. 10.11646/zootaxa.1453.1

[CR16] Hoch H (2013) Diversity and evolution of the Southeast-Asian plant-hopper taxon Bennini (Hemiptera, Cixiidae). Nova Supplementa Entomologica (Senckenberg Deutsches Entomologisches Inst Müncheberg Germany) 23:1–296

[CR19] Hoch H, Wessel A, Asche M, Baum D, Beckmann F, Bräunig P, Ehrig K, Mühlethaler R, Riesemeier H, Staude A, Stelbrink B, Wachmann E, Weintraub P, Wipfler B, Wolff C, Zilch M (2014) Non-sexual abdominal appendages in adult insects challenge a 300 million year old Bauplan. Curr Biol 24:R16–R17. 10.1016/j.cub.2013.11.040 24405669 10.1016/j.cub.2013.11.040

[CR18] Hoch H, Sendra A, Montagud S, Teruel S, Lopes Ferreira R (2021) First record of a cavernicolous Kinnaridae from the old world (Hemiptera, Auchenorrhyncha, Fulgoromorpha, Kinnaridae, Adolendini) provides testimony of an ancient fauna. Subterr Biol 37:1–26. 10.3897/subtbiol.37.60483

[CR17] Hoch H, López H, Naranjo M, Aguín-Pombo D, Oromí P (2025) Endless forms most wonderful: four new cavernicolous planthopper species (Hemiptera, Fulgoromorpha, Cixiidae and Meenoplidae) from the Canary Islands. Subterr Biol 51:61–101. 10.3897/subtbiol.51.144111

[CR21] Holzinger WE, Kunz G (2006) A new genus and species of Bennarellini from Costa Rica (Hemiptera: Fulgoromorpha: Cixiidae). Zootaxa 1353:53–61

[CR20] Holzinger WE, Holzinger I, Egger J (2013) A new genus, *Loisirella*, and two new species of Bennarellini from Ecuador (Hemiptera: Auchenorrhyncha: Fulgoromorpha: Cixiidae). Acta Musei Morav Sci Biol Brno 98:143–153

[CR22] Jackson CW, Hunt E, Sharkh S, Newland PL (2011) Static electric fields modify the locomotory behaviour of cockroaches. J Exp Biol 214:2020–2026. 10.1242/jeb.053470 21613518 10.1242/jeb.053470

[CR23] Keil TA (2012) Sensory cilia in arthropods. Arthropod Struct Dev 41:515–534. 10.1016/j.asd.2012.07.001 22814269 10.1016/j.asd.2012.07.001

[CR24] Khan SA, Khan KA, Kubik S, Ahmad S, Ghramh HA, Ahmad A, Skalicky M, Naveed Z, Malik S, Khalofah A, Aljedani DM (2021) Electric field detection as floral cue in hoverfly pollination. Sci Rep 11:18781. 10.1038/s41598-021-98371-4 34548579 10.1038/s41598-021-98371-4 PMC8455601

[CR25] Liebenberg K (1955) Untersuchungen über die postembryonale Entwicklung der äußeren Merkmale und ein neues larvales Haarsinnesorgan von *Callipygona pellucida* F. (Homoptera, Cicadina). Doctoral Thesis, Freie Universität Berlin

[CR26] Newland PL, Hunt E, Sharkh SM, Hama N, Takahata M, Jackson CW (2008) Static electric field detection and behavioural avoidance in cockroaches. J Exp Biol 211:3682–3690. 10.1242/jeb.019901 19011207 10.1242/jeb.019901

[CR27] Shcherbakov DE (2007) An extraordinary new family of cretaceous planthoppers (Homoptera: Fulgoroidea). Russ Entomol J 16:139–154

[CR28] Sulc K (1928) Die Wachsdrüsen und ihre Produkte bei den Larven der Cixiinen (Homoptera). Biol Sp Vysoke Sk Zwerolekaoske Brno 7:1–32

[CR29] Sutton GP, Clarke D, Morley EL, Robert D (2016) Mechanosensory hairs in bumblebees (*Bombus terrestris*) detect weak electric fields. Proc Natl Acad Sci U S A 113:7261–7265. 10.1073/pnas.1601624113 27247399 10.1073/pnas.1601624113 PMC4932954

[CR30] Szwedo J (2007) Nymphs of a new family Neazoniidae fam. n. (Hemiptera: Fulgoromorpha: Fulgoroidea) from the lower cretaceous Lebanese amber. Afr Invertebr 48:127–143

[CR31] Thurm U (1983) Fundamentals of transduction mechanisms in sensory cells. Biophysics. Springer, Berlin

[CR32] Tomás A, Rebelo MT, Valkenburg T, Darby M, da Pereira Fonseca I (2018) An association between the featherwing beetle *Ptiliolum fuscum* (Erichson) (Coleoptera: Ptiliidae) and the Eurasian griffon *Gyps fulvu*s (Hablizi) (Accipitriformes: Accipitridae) – first report of a phoretic interaction between beetles and birds? Coleopt Bull 72:662. 10.1649/0010-065X-72.4.662

[CR33] Viegas EFG, Ale-Rocha R (2019) A review of the Neotropical genus *Amazobenna* Penny, 1980 with description of a new species and description of the male of *Amazobenna reticulata* Penny, 1980 (Hemiptera: Fulgoromorpha: Cixiidae). Zootaxa. 10.11646/zootaxa.4577.3.9 31715715 10.11646/zootaxa.4577.3.9

[CR34] Viegas EFG, Ale-Rocha R (2022) Study of the Neotropical genus *Bennarella* Muir, 1930 with description of six new species (Hemiptera: Fulgoromorpha: Cixiidae). Zootaxa 5124:155–187. 10.11646/zootaxa.5124.2.3 35391130 10.11646/zootaxa.5124.2.3

[CR35] Waloff N, Jervis MA (1987) Communities of parasitoids associated with leafhoppers and planthoppers in Europe. Adv Ecol Res 17:281–376. 10.1016/S0065-2504(08)60248-2

[CR36] Wilson MR (1989) The planthopper family Achilixiidae (Homoptera, Fulgoroidea): a synopsis with a revision of the genus *Achilixius*. Syst Entomol 14:487–506. 10.1111/j.1365-3113.1989.tb00299.x

[CR37] Yang C-T, Yeh W-B (1994) Nymphs of Fulgoroidea (Homoptera: Auchenorrhyncha) with descriptions of two new species and notes on adults of Dictyopharidae. Chin J Entomol Spec Publ 8:1–189

