# Supplementary material for: Electrosensitivity in planthoppers (Insecta: Hemiptera: Auchenorrhyncha: Fulgoromorpha)
Source: J Comp Physiol A Neuroethol Sens Neural Behav Physiol. 2026 Jan 7;212(3):487–99. doi: 10.1007/s00359-025-01790-1 (PMC13198465; doi:10.1007/s00359-025-01790-1)
Supplement: Supplementary file 1 — Supplementary Material 1 [file 359_2025_1790_MOESM1_ESM.pdf]

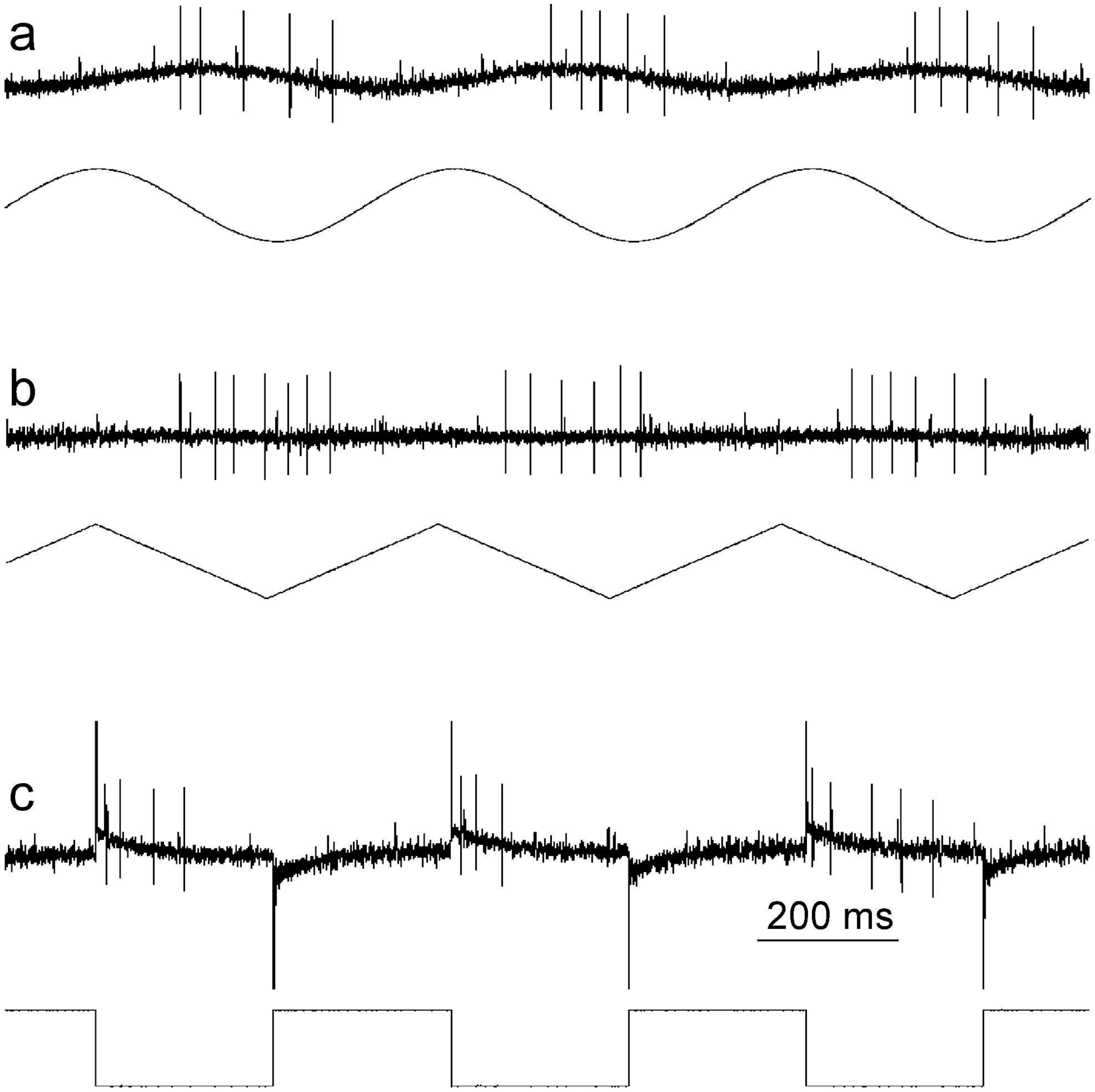


Supplementary Figure 1

Response of a sensory pit afferent to electric fields modulated at 2 Hz in sinusoidal (**a**), triangular (**b**) and rectangular (**c**) fashion. The pit was located on the mesothoracic tergum and the stimulating wire was positioned approximately 800 µm distant above the tergite of the third abdominal segment. The stimulus amplitude was 2V peak-peak. All three panels show unfiltered recordings. This explains the baseline modulations in **a** and **c,** and the stimulus artifacts in **c**.
