# Supplementary material for: Electrosensitivity in planthoppers (Insecta: Hemiptera: Auchenorrhyncha: Fulgoromorpha)
Source: J Comp Physiol A Neuroethol Sens Neural Behav Physiol. 2026 Jan 7;212(3):487–99. doi: 10.1007/s00359-025-01790-1 (PMC13198465; doi:10.1007/s00359-025-01790-1)
Supplement: Supplementary file 2 — Supplementary Material 2 [file 359_2025_1790_MOESM2_ESM.pdf]

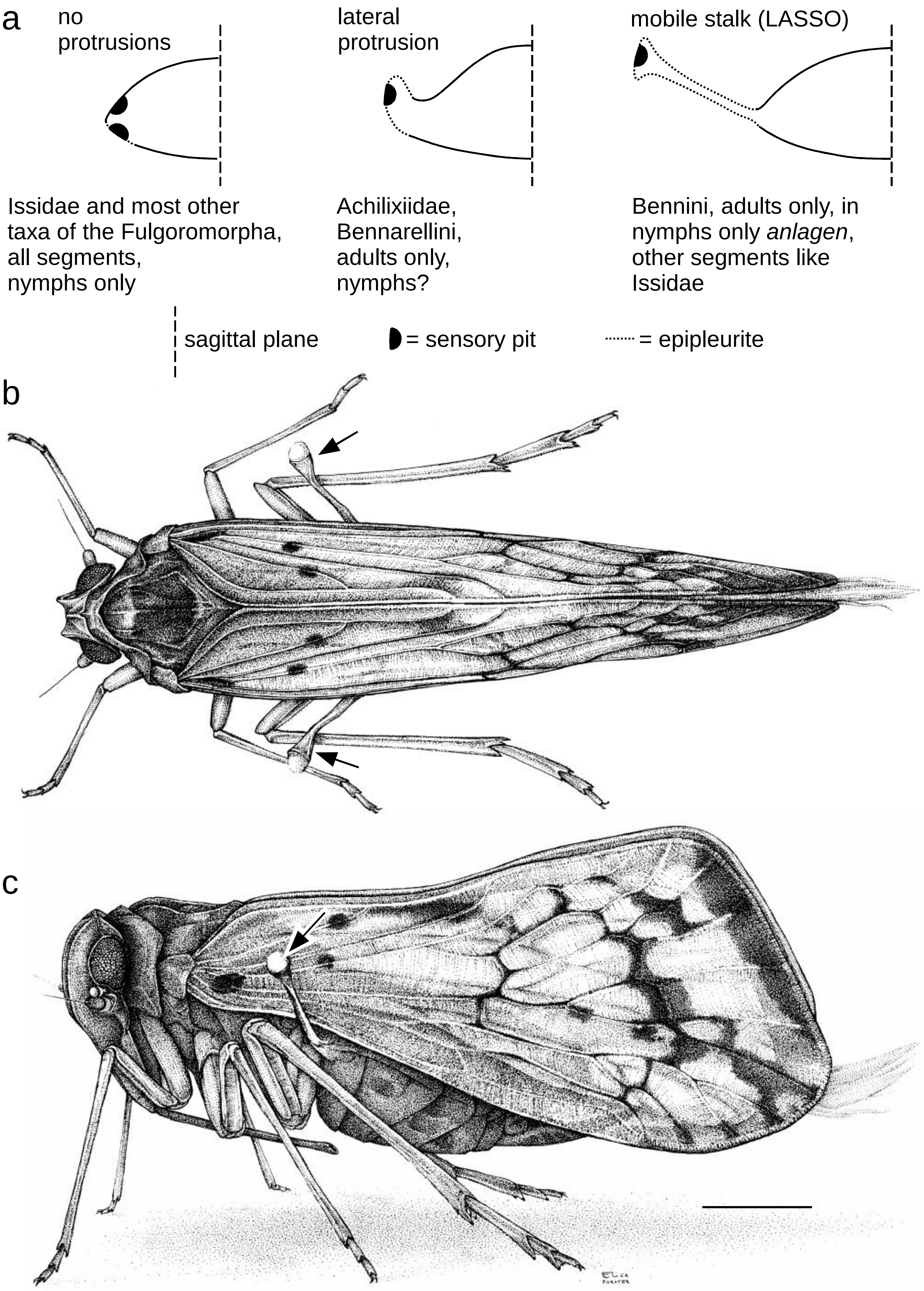
a
no
protrusions
lateral
protrusion
mobile stalk (LASSO)
Issidae and most other
taxa of the Fulgoromorpha,
all segments,
nymphs only
Achilixiidae,
Bennarellini,
adults only,
nymphs?
Bennini, adults only, in
nymphs only *anlagen*,
other segments like
Issidae
sagittal plane
= sensory pit
= epipleurite
b
c

Supplementary Figure 2

Lateral displacement of sensory pits in abdominal segments of various Fulgoromorpha taxa. **a** Schematic cross sections of abdominal hemisegments illustrating these lateral displacements: In the Issidae (left), and most other taxa of the Fulgoromorpha, sensory pits occur in nymphs only. There is no modification of abdominal segments in adults. In adults of some species of the Achilixiidae and the Bennarellini (middle), some abdominal segments form lateral bulges that bear sensory pits (Achilixiidae: one or two protrusions between segments 3 and 5; Bennarellini: one protrusion spanning segments 4 and 5). In adult Bennini (right), sensory pit-like structures are located at the distal end of the so-called LASSO (lateral sensory and secretory organs; Hoch et al. 2014), slender stalk-like, mobile appendages (formed between segments 3 and 4). These appendages are not yet present in the nymphs that otherwise look like the nymphs of the Issidae. Nothing is known about the distribution of sensory pits in nymphs of Achilixiidae and Bennarellini. **b** and **c** Habitus of an adult female of a Bennini species (*Benna capitulata* Walker, 1857), dorsal (**b**) and lateral (**c**) view. The arrows point at the LASSO. Scale: 1 mm (Drawings by Elisa Forster with permission)
